# Risk and resilience in eating disorders: differentiating pathways among psychosocial predictors

**DOI:** 10.1186/s40337-024-01023-x

**Published:** 2024-05-21

**Authors:** Maria Bazo Perez, Leslie D. Frazier

**Affiliations:** Department of Psychology, Florida International University, 11200 SW 8th Street, Miami, FL 33199 USA

**Keywords:** Eating disorders, Disordered eating, Perfectionism, Anxiety sensitivity, Emotion dysregulation, Body dissatisfaction, Structural equation model, Subscales

## Abstract

**Objective:**

Eating disorders (EDs) represent a rising global health concern. The current study takes a multivariate approach to examine psychological (i.e., perfectionism, anxiety sensitivity [AS], emotion dysregulation) and sociocultural factors (i.e., body dissatisfaction) that may relate to risk and resilience in EDs.

**Methods:**

Participants were 698 undergraduate students (*M*_*age*_ = 21, *SD*_*age*_ = 4.02), mainly female (71%) and Hispanic (61.6%), who participated in an online survey assessing perfectionism, emotion dysregulation, AS, body dissatisfaction, and eating behaviors.

**Results:**

The results from structural equation model analyses revealed differential associations with disordered eating (DE) outcomes. Self-oriented perfectionism and dysmorphic appearance concerns were associated with increased dieting/carb restriction, desire for thinness, and binging tendencies. Specifically, emotional nonacceptance and lack of emotional awareness showed associations with elevated risk for dieting/carb restriction and purging tendencies, respectively. Conversely, lack of emotional clarity showed a protective pathway to these risk behaviors. Anxiety sensitivity cognitive concerns related to higher purging tendencies, while AS social concerns related to lower purging and binging tendencies.

**Discussion:**

Findings highlight the differential pathways of psychosocial risk and resilience for EDs. Subscales of emotional dysregulation and AS showed risk as well as resilience associations with DE outcomes. This information is key for advancing transdiagnostic prevention and intervention to reduce the rising rates of EDs.

## Abstract (plain language summary)

Eating disorders are rising worldwide at alarming rates. We know their development is complex involving multiple factors, but the specific contributions of different factors are not well understood. This study demonstrates differential pathways of risk and resilience among psychosocial factors (i.e., perfectionism, emotion dysregulation, anxiety sensitivity, and body dissatisfaction) and eating behaviors and cognitions. Facets of perfectionism and body dissatisfaction were associated with unhealthy eating behaviors like dieting and bingeing. However, different aspects of emotional dysregulation and anxiety sensitivity were linked to maladaptive eating behaviors, but others seemed to protect against risky eating behaviors. This information is crucial for creating more effective prevention and treatment strategies for eating disorders.

## Introduction

Eating disorders (EDs) are mental disorders that have among the highest mortality rates [1, 2]. Since 2013, the worldwide prevalence of EDs increased from 3.5 to 7.8% [3], now nearly 10% of Americans will have an ED at some point in their lives [4]. The etiology of EDs is complex and multidetermined, encompassing several biopsychosocial determinants [5, 6]. A recent review on risk factors proposed that different biological (e.g., gut bacteria), psychological (e.g., perfectionism, anxiety sensitivity [AS], emotional dysregulation), and sociocultural factors (e.g., body dissatisfaction) are involved in the development of EDs [7]. Research on the dynamic, interactive, and co-occurring risk factors suggests the importance of a multivariate approach that articulates the coactive influences of multidimensional factors [8, 9]. However, this approach has largely been overlooked in favor of understanding and addressing individual risk factors [10–12]. We argue that specificity and effectiveness in transdiagnostic prevention/intervention of EDs is dependent upon examining the differential pathways of latent factors on outcomes. The present research adopts a multivariate (i.e., multiple outcomes) and multidimensional (i.e., multiple predictors or dimensions) framework to identify the specific associations and unique risk and resilience pathways among different dimensions of psychosocial factors (i.e., perfectionism, AS, body dissatisfaction, and emotional dysregulation) with ED outcomes. We examine these associations in a sample of predominately Hispanic/Latinx participants. Eating pathologies are rising among understudied communities, including racial and ethnic minorities [13, 14]. Additionally, prevalence rates of EDs within the Hispanic and Latinx communities are comparable or even higher than non-Latinx Whites [15, 16]. Studies indicate that individuals from diverse ethnic backgrounds might perceive and experience EDs in unique ways, and that symptoms often linked with one ethnic group might not apply to others within different ethnic minorities [13]. Research on risk factors for EDs among ethnic minorities is needed. Ultimately, given the severe health consequences of EDs, the rising rates across underrepresented groups, and the increasing rates of minorities in U.S. society [17], it is imperative to understand the factors that put individuals at risk for eating pathologies.

### Eating behaviors

Eating disorders exist on a spectrum.^1^ Eating disorders are characterized by persistent disturbances of eating-related behaviors (e.g., restriction, binging, or purging), that may lead to substantial impairment in physical health and/or psychosocial functioning [18]. Disordered eating (DE) represents a subthreshold condition in which attitudes and behaviors may be present, but not at a diagnostic level [19]. Thus, the frequency and severity of the maladaptive eating behaviors mark the distinction between EDs and DE [20]. Disordered eating is associated with emotional distress and impairment [21], and represents the most important predictor of EDs [22]. In particular, DE behaviors (e.g., eating in secret, laxative abuse, fear of losing control over eating) are predictive of future EDs [23]. As ED prevalence rises, so do rates of DE [3].

### Risk factors of eating disorders

#### Perfectionism

Perfectionism, a core feature of severe EDs [24], is predictive of both the onset and maintenance of the disorder [25–27]. Longitudinal research showed that higher perfectionism scores at pretest were associated with meeting diagnostic criteria for EDs 1 year later [28].

Perfectionism, a multidimensional^2^ construct comprising both intra- and interpersonal components, is related to the pursuit and over-evaluation of high standards, despite adverse consequences [29].

As a transdiagnostic risk factor, perfectionism is thought to interact with other risk factors predisposing and maintaining the ED [30]. Both individuals with EDs and individuals high in DE have higher levels of perfectionism than controls [31, 32]. Individuals with EDs and those who are not diagnosed with an ED, perfectionism is a predictor of core ED symptomology, such as body dissatisfaction and the drive for thinness [26, 33, 34]. Specific facets of perfectionism manifest differentially in ED symptomology, for example, higher scores on self-oriented and socially-perceived are linked with higher body dissatisfaction and higher reporting of concerns about weight and body shape [30, 35, 36].

Perfectionism is linked to social anxiety associated with one’s appearance, and the fear of being negatively judged by others, and these associations are related to increased DE [37]. Worry about one’s imperfections is a strong predictor of DE among women [38]. Individuals with elevated ED risk hold high standards regarding their eating and body appearance which may lead them to greater perfectionism in these areas [39]. Taken together, perfectionism is considered an important risk factor to be addressed in the prevention/intervention of EDs [40].

#### Emotion dysregulation

Emotion regulation refers to one’s ability to effectively manage and respond to emotional experiences or situations, including the processes used to control, evaluate, and adjust one’s emotional responses [41, 42]. When emotions are not regulated efficiently or successfully, emotion dysregulation occurs (i.e., the rigid and maladaptive reliance on emotion regulation strategies, like rumination, avoidance, suppression, aggression, venting, denial) [43]. There is a strong relationship among emotion regulation difficulties and many clinical outcomes [43]. In fact, emotion dysregulation may be the “hallmark of psychopathology” [44].

Difficulties with emotion regulation are related to ED symptomatology development, maintenance, and outcomes [45]. Individuals diagnosed with EDs show higher levels of global emotion dysregulation than controls [46, 47]. Meta-analytic evidence suggests that high levels of negative emotionality aggravate the risk for eating pathology [12]. Symptomology of EDs (e.g., dietary restriction, excessive exercise, or binging and purging), may represent maladaptive attempts to regulate negative emotional states [48, 49]. Lavender et al. [50] found evidence that deficits in adaptive emotion regulation skills, emotional awareness, emotional avoidance, impulse control, and distress tolerance difficulties were associated with anorexia nervosa. Research shows that individuals with EDs are more likely to use maladaptive strategies such as self-destructive behaviors, avoiding emotional experiences, excessive focusing on an emotion (rumination), suppression of desires or negative affect, and ineffective coping techniques [51].

Problems in regulating emotions, including emotional management and disengagement, may lead some individuals with EDs to be unable to shift or disengage attention away from dysfunctional thoughts, which can intensify their negative emotions [51]. The absence of effective behavioral strategies can result in further emotion dysregulation. Consistent with the transactional model of emotion dysregulation, individuals at risk for EDs may over time accumulate a history of invalidating responses regarding their inner experiences (e.g., hunger and satiety, body image, emotional reactions to eating). Haynos and Fruzzetti [51] suggest that as individuals increasingly immerse in their ED (e.g., by fixating on food or body cues), their emotional arousal amplifies across various emotionally significant contexts. What this means is that the maladaptive behavioral responses or strategies that those with EDs use may help them alleviate their emotional arousal, and this may negatively reinforce using these maladaptive strategies again and again.

#### Anxiety sensitivity

Anxiety sensitivity, or the fear of fear, is a mental health vulnerability that relates to emotion dysregulation [52, 53]. Anxiety sensitivity is the fear of experiencing anxiety-related bodily sensations and arises from the misconception that these sensations carry negative physical, cognitive, or social consequences [53–55]. In other words, AS is the tendency to see the experiences of anxiety as highly problematic and aversive [56]. A multidimensional construct, AS has three dimensions^3^: physical, cognitive, and social concerns [57]. People who experience high levels of AS often amplify and misinterpret bodily sensations and anxiety symptoms [58]. They are also more likely to overestimate and exaggerate the negative consequences of anxiety and try to avoid anxiety-provoking situations [55, 59]. Emotion dysregulation in individuals with anxiety can manifest through amplified intensity of emotions, negative reactivity, and poor understanding of one’s emotions as well as a maladaptive emotional response [60].

High levels and more intense experiences of negative affect represent a shared vulnerability with AS in the development and maintenance of EDs and other internalizing disorders [47, 61, 62]. Anxiety sensitivity is positively correlated with the drive for thinness and the severity of bulimic symptoms [63]. These findings reinforce that anxiety is often perceived to carry unacceptable negative consequences, to be avoided or repressed through ED behaviors [46, 47]. The experiential avoidance of emotions further reinforces negative expectancies about emotion, maintaining the avoidance patterns and ED symptomology [55, 64, 65]. Espel-Huynh et al. [66] found that experiential avoidance mediated the relationship between the social dimension of AS and eating pathology. Among college students, AS has shown a positive association with global ED symptom severity, with higher levels of AS found in individuals who also reported higher DE [67]. However, recent research by Bazo Perez et al. [53] on the associations among AS subscales and ED risk in a large sample of young adults found that higher AS cognitive concerns were associated with higher EDs symptoms, while higher AS social concerns were associated with fewer EDs symptoms, showing a potential protective pathway of this dimension of AS.

#### Body dissatisfaction

Body dissatisfaction refers to the negative evaluation of one’s body appearance, specific body features, or other feelings related to body image [68]. Like self-discrepancy theory [69, 70], body dissatisfaction emerges from a discrepancy between the perceived and ideal body images. It is difficult for women to avoid being pressured to internalize the “thin ideal,” and research shows that a consequence, self-worth is highly dependent on how others view them, directly connecting body image satisfaction to well-being [71].

Extensive research suggests that excessive dieting in response to body dissatisfaction may lead to an increased risk of developing and maintaining ED pathologies [72, 73]. The DSM-5 includes body image disturbance (i.e., body dissatisfaction) as a diagnostic criterion for anorexia and bulimia nervosa [18]. Research shows that body dissatisfaction is strongly associated with DE pathology [74]. Negative perceptions about one’s body may contribute to experiencing the body as separate from the self, leading to self-destructive behaviors [75, 76]. The mechanism through which higher levels of body dissatisfaction result in a higher likelihood of ED onset has been identified as an attempt for emotional regulation [77].

### The proposed research study

**Fig. 1:**
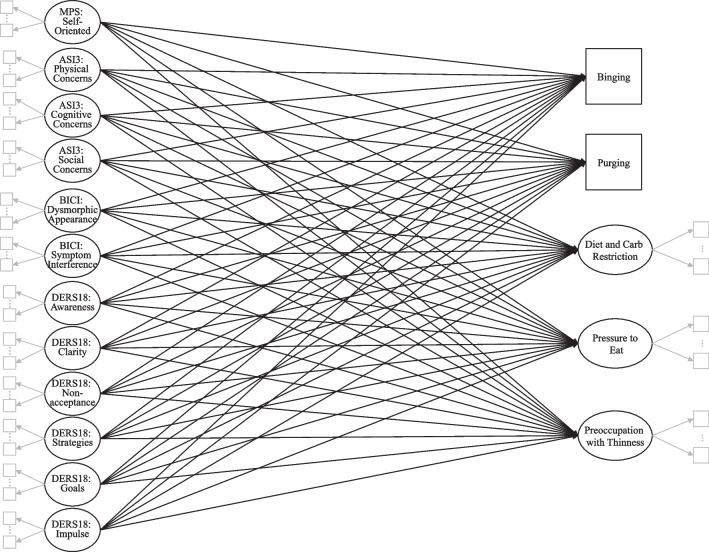
Path diagram of the latent variable regression model. *Note:* For ease of reading, this diagram omits: **a** exogenous variances and covariances, **b** item residuals, **c** endogenous disturbances, and **d** disturbance variances and covariances. Ellipses, ⋮, between factor model indicators suggest that additional indicators may load on each factor model (but are omitted to conserve space). Solid black versus solid grayscale lines are used to visually distinguish the regression model from the measurement models, respectively

While extensive research has delved into identifying psychosocial risk factors for EDs individually, to our knowledge, no study has thoroughly investigated, at the subscale level, how established risk factors associate with various eating cognitions and behaviors within a culturally diverse sample. Thus, our study aimed to identify patterns of ED risk within a predominately Hispanic sample, which is crucial for providing more effective prevention and intervention profiles. Specifically, we assessed how the established risk factors for EDs (i.e., perfectionism, emotion dysregulation, AS, and body dissatisfaction) may associate with different eating outcomes. We predicted that these psychosocial factors would differentially associate with DE outcomes, and to test this prediction, we fit a Structural Equation Model (SEM) displayed in the path diagram in Fig. 1. We examined the associations at the subscale level to understand the uniqueness of the different paths more comprehensively. We conducted Confirmatory Factor Analyses (CFAs) to determine the relationships among latent variables and their indicators, and to determine the integrity of the standardized measures as well as the predictive value of the constructs that compose the factors.

In contrast to much of the past research on eating pathologies, which has focused on studying different psychosocial risk factors in isolation, the present study addressed these factors within a single complex model, controlling for the influences of other factors.

## Methods

### Participants

This research used existing data collected from 1014 undergraduate students at a large urban public university in Florida, United States. The majority of the sample was female (71%), with an average age of 21 (*SD* = 4.02). Participants self-identified their racial and ethnic backgrounds as follows: Hispanic (61.6%), African American (9.6%), White Non-Hispanic (7.4%), South Asian (e.g., Indian, Pakistani, 1.1%), Asian/Asian American (1.4%), Native American (0.1%), Other (4.9%), and no response (14%). The sample's demographic composition closely mirrored the characteristics of the major urban public research university and its surrounding community.

### Measures

#### Perfectionism

Perfectionism was assessed using the Hewitt Multidimensional Perfectionism Scale (MPS) [78]. This 45-item Likert-type scale measures participants’ feelings of perfectionism on three dimensions: (1) self-oriented perfectionism; (2) other-oriented perfectionism; and (3) socially perceived perfectionism.

Preliminary analyses showed that the other-oriented and socially perceived subscales exhibited notably low/close to zero correlations with other model constructs (correlations ranged from 0.04 to 0.23 for the others-oriented perfectionism subscale and the DE outcomes; and ranged from 0.04 to 0.28 for the socially perceived perfectionism subscale and the DE outcomes). When included they significantly reduced the overall fit and created poor fit levels in our model. Therefore (see also [30, 35, 36]), we included only the self-oriented subscale. In our sample, the average total score on the MPS was 187.12 (*SD* = 25.21), and the mean score on the self-oriented perfectionism subscale was 71.88 (*SD* = 13.99). The internal reliability of the MPS was calculated at α = 0.84, and the self-oriented perfectionism subscale achieved an α = 0.88, both indicating good internal consistency (after item 12 was deleted for low and negative correlations with other items).

#### Emotion dysregulation

Emotional dysregulation was assessed using the Brief Version of the Difficulties in Emotion Regulation Scale (DERS-18) [79]. This 18-item Likert-type self-report measure evaluates participant’s modulation of arousal, awareness, understanding, and acceptance of emotions across six subscales: (1) non-acceptance of emotional responses; (2) difficulties in engaging in goal-directed behaviors; (3) impulse control difficulties; (4) lack of emotional awareness; (5) limited access to emotion regulation strategies; and (6) lack of emotional clarity. Total scores range from 18 to 90, with higher scores representing greater difficulties in emotion regulation. In our sample, the average total score on the DERS-18 was 38.60 (*SD* = 13.37). The internal reliability of the DERS-18 total score was calculated at α = 0.92, indicating excellent internal consistency. Furthermore, the subscales demonstrated good to excellent internal reliability: non-acceptance α = 0.91, goals α = 0.91, impulse α = 0.89, awareness α = 0.82, strategies α = 0.85, and clarity α = 0.85.

#### Anxiety sensitivity

Anxiety sensitivity was measured using the Anxiety Sensitivity Index (ASI-3) [57]. The ASI-3 is an 18-item Likert-type scale measuring concerns regarding arousal-related sensation across three subscales: (1) physical concerns; (2) cognitive concerns; and (3) social concerns. Total scores range from 0 to 72, with higher scores reflecting higher AS. In our sample, the average total score on the ASI-3 was 22.22 (*SD* = 16.65), which indicates relatively low AS. Internal reliability for the ASI-3 total score was calculated at α = 0.95, indicating excellent internal consistency. Moreover, the subscales also demonstrated strong internal reliability: cognitive concerns α = 0.92, physical concerns α = 0.89, and social concerns α = 0.84.

#### Body dissatisfaction

Body shape concerns were measured using the Body Image Concern Inventory (BICI) [80]. This is a 19-item self-report questionnaire that asks participants to answer questions regarding how often they experience the feeling or execute the behavior described. This Likert-type scale divides items into two subscales: (1) dysmorphic appearance concern; and (2) symptom interference. Overall scores range from 19 to 95, with higher scores representing greater dissatisfaction with one's body image or appearance. In our sample, the average total score on the BICI was 49.13 (*SD* = 17.67). Internal reliability of the BICI was calculated at α = 0.96, indicating excellent internal consistency. Additionally, the subscales also demonstrated strong internal reliability: dysmorphic appearance concern subscale α = 0.94, and symptom interference α = 0.91.

#### Disordered eating

To assess risk for DE or EDs, the Eating Attitudes Test-26 (EAT-26) [81] was used. The EAT-26 provides scores on three subscales: (1) dieting; (2) bulimia and food preoccupation; and (3) oral control. Participants respond on a 6-point Likert scale ranging from 1 (*never*) to 6 (*always*). Global scores range from 0 to 78, with 20 as the original cutoff, scores greater than 20 indicating a higher risk of developing an ED, and scores below this cutoff indicate a lower risk [82]. In our sample, the average total score was 9.68 (*SD* = 9.15), close to the recent cutoff of 11 and above for risk of overweight, bulimic, and binge-purge symptoms established in recent research [83–85]. In our sample n = 97 participants scored above 20, and n = 232 scored above 11.

Despite its extensive use, there is an emerging body of literature questioning the factor structure of the EAT-26, as the three-factor structure—originally developed in a sample of women diagnosed with anorexia nervosa—does not seem to perform the same way for non-clinical and non-WEIRD (Western Educated Industrialized Rich, and Democratic) samples. Different factorial structures have been reported in populations including non-clinical samples [86], different cultures and ethnic backgrounds [87–89], or across genders [90]. Therefore, the factor structure of the EAT-26 was analyzed in this sample (see also [53]).^4^ Results from the Exploratory Factor Analyses (EFA) and CFA suggested a four-factor structure: (1) diet foods/carbs restriction (comprising items 7, 16, and 17); (2) pressure to eat (including items 8, 13, and 20); (3) desire for thinness (containing items 1, 11, 14); and (4) purging tendencies (measured by a single-item indicator of item 25). Additionally, we introduced the EAT-26 item 4 as a single-item indicator of binging behaviors, since we considered that examining this particular type of DE behavior was significantly relevant. The internal reliability of the global 4-factor EAT-26 has been calculated at α = 0.84, indicating good internal reliability (deleting items 19 “I Display self-control around food” and 26 “I Enjoy trying new rich foods” for having low, close to zero correlations with other items raised the internal reliability to α = 0.86). Looking at the subscales, diet foods/carbs reduction achieved α = 0.71, pressure to eat α = 0.72, and desire for thinness α = 0.82, indicating good to acceptable internal reliability.

### Procedure

The data in this study were part of a large cross-sectional dataset on weight-related concerns and health behaviors in college students. This study had IRB approval. Potential participants were recruited through the SONA human subjects recruitment online system and received course credit for participation. After providing informed consent, participants anonymously and confidentially completed a series of different surveys through online survey software (i.e., Qualtrics).

### Data analytic approach

To verify/confirm the underlying latent factor structure of all the measured variables, CFAs were performed. We took a latent variable approach, assuming participants’ observed scores as imperfect indicators of the true level of constructs. This approach allows to take measurement error into account, obtaining more precise regression coefficients and unbiased estimates. Results from the CFAs dictated the factor structure tested in the SEM model and provided construct validity of measurement models. The fit of the SEM model^5^ was assessed and the pathways of perfectionism, AS, emotional dysregulation, and body dissatisfaction to determine if our structural model illuminated differential associations with DE outcomes. To appropriately account for the categorical nature of the measured variables (i.e., all items were Likert-type), all models were estimated using the weighted least squares mean and variance adjusted (*WLSMV*) estimation in the lavaan Package [91] in R [92].

### Treatment of missing data

The initial sample size of this dataset was *N* = 1014. After eliminating participants who failed the attention check, the sample resulted in *n* = 948. Of these, 153 individuals had missing data across all MPS, ASI-3, BICI, DERS-18, and EAT-26 items and were dropped from the analyses for not providing information to the full information maximum likelihood (*FIML*) [93]. The resulting sample was *n* = 795. However, the *WLSMV* estimation method used in the CFA/SEM analyses required complete data, leading to the elimination of further cases with a final sample of *n* = 698.

## Results

### Measurement model fit

Table 1 displays the fit statistics for the CFAs of each subscale and scale according to the original established factor structures (except for the EAT-26). All measurement models displayed good fit across most indices, except the perfectionism subscale of the MPS, which exhibited poor to acceptable fit. The Comparative Fit Indices (*CFI*) [94] for all models were above the cutoff of > 0.95 (except the MPS subscale 0.92, indicating approximately 92% improvement in fit -reduction in approximation error- over the baseline model). The Tucker Lewis Index (*TLI*) [95] also demonstrated good fit for all models being above the cutoff > 0.95 (except the MPS subscale 0.91, indicating that this model explained approximately 91% of the population covariances, and reduced around 91% in the misfit of the baseline model approximation error). The Root Mean Square Error of Approximation (*RMSEA*) [96, 97] showed results for all models above the cutoff of < 0.05 (DERS-18 right at the cutoff), indicating increases in the standardized covariance residuals per degrees of freedom due to approximation error, thus suggesting misfit. The Standardized Root Mean Residual (*SRMR*) [98], was below the cutoff of < 0.08 across all models (except the EAT-26 4-factor model, indicating an average correlation residual of 0.09).

**Table 1:** Model fit indices

	MPS perfectionism	ASI-3 physical	ASI-3 cognitive	ASI-3 Social	BICI dysmorphic appearance	BICI symptom interference	DERS-18 6-factor	EAT-26 4-factor	Latent variable regression model
Chi-square	1105.22	92.192	1126.13	48.47	780.09	83.194	315.778	132.13	6592.379
*df*	65	9	9	9	54	14	120	38	3025
*p*	< .001	< .001	< .001	< .001	< .001	< .001	< .001	< .001	< .001
CFI	0.92	0.99	0.99	0.99	0.97	0.99	0.99	0.97	0.95
TLI	0.91	0.98	0.98	0.99	0.97	0.99	0.99	0.96	0.95
RMSEA	0.15	0.11	0.13	0.08	0.13	0.08	0.05	0.06	0.04
90% CI	[0.14, 0.15]	[0.09, 0.13]	[0.11, 0.15]	[0.06, 0.10]	[0.12, 0.14]	[0.05,0.10]	[0.04, 0.05]	[0.05, 0.07]	[0.04, 0.04]
*p*_*close*_	< .001	< .001	< .001	0.019	< .001	0.002	0.85	0.156	1
SRMR	0.07	0.03	0.03	0.03	0.05	0.03	0.03	0.09	0.06

All models were fit using WLSMV estimation in lavaan Package. Fit statistics are reported from the robust column of output

### Structural model fit

The right-most column in Table 1 shows the absolute and comparative fit indices for our structural model. Indices denoted good model fit: the *CFI* and *TLI* were right at the cutoff of > 0.95, and the *RMSEA* and *SRMR* were both below the cutoffs of > 0.05 and > 0.08 respectively.

### Latent variable regression results

Table 2 presents the correlations between our model predictors and outcomes. Most pairings yielded low to moderate correlations, with the highest correlations achieved by the two BICI subscales and the desire for thinness EAT-26 outcome.

**Table 2:** Model predictor and outcome correlations

	1	2	3	4	5	6	7	8	9	10	11	12	13	14	15	16	17
1. Diet food/carb reduction																	
2. Pressure to eat	**0.148**																
3. Desire for thinness	**0.366**	0.066															
4. MPS Perfectionism	**0.121**	**0.114**	**0.210**														
5. ASI3 Physical concerns	− 0.055	0.048	**0.218**	**0.155**													
6. ASI3 Cognitive concerns	− 0.014	**0.140**	**0.296**	**0.196**	**0.758**												
7. ASI3 Social concerns	− 0.037	**0.133**	**0.297**	**0.241**	**0.766**	**0.820**											
8. BICI Dysmorphic	**0.091**	**0.126**	**0.669**	**0.239**	**0.388**	**0.483**	**0.518**										
9. BICI Symptom	0.039	**0.122**	**0.602**	**0.207**	**0.410**	**0.518**	**0.537**	**0.835**									
10. DERS18 Awareness	− **0.079**	0.069	0.068	− 0.042	**0.122**	**0.207**	**0.209**	**0.205**	**0.294**								
11. DERS18 Clarity	− **0.095**	**0.158**	**0.307**	**0.126**	**0.352**	**0.514**	**0.460**	**0.494**	**0.545**	**0.457**							
12. DERS18 Nonacceptance	**0.088**	**0.169**	**0.283**	**0.174**	**0.303**	**0.450**	**0.454**	**0.409**	**0.465**	**0.179**	**0.496**						
13. DERS18 Strategies	− 0.016	**0.121**	**0.415**	**0.162**	**0.443**	**0.595**	**0.528**	**0.565**	**0.625**	**0.265**	**0.637**	**0.645**					
14. DERS18 Goals	0.010	**0.143**	**0.290**	**0.117**	**0.297**	**0.432**	**0.438**	**0.446**	**0.429**	**0.118**	**0.470**	**0.560**	**0.705**				
15. DERS18 Impulse	0.036	**0.111**	**0.316**	**0.117**	**0.278**	**0.469**	**0.359**	**0.403**	**0.448**	**0.201**	**0.503**	**0.595**	**0.748**	**0.605**			
16. EAT26_25 Purging	**0.087**	**0.124**	**0.176**	0.001	**0.087**	**0.116**	0.076	**0.145**	**0.156**	**0.091**	**0.087**	0.065	**0.134**	0.055	**0.112**		
17. EAT26_4 Binging	**0.086**	− 0.031	**0.374**	0.017	**0.138**	**0.159**	**0.103**	**0.289**	**0.308**	0.064	**0.179**	**0.137**	**0.253**	**0.131**	**0.194**	**0.206**	

Correlations were estimated using the what = “cor.all” argument in the lavInspect() function in lavaan. Bolded entries indicate significant results at or below the .05 level

Table 3 shows the standardized parameter estimates for our model (see Fig. 1). Note that not all key predictors included in our model were significant.

**Table 3:** Standardized latent regression results of EAT-26 outcomes predicted by subscales of perfectionism, anxiety sensitivity, body dissatisfaction, and emotion dysregulation

	Latent variable model					
	*Std. Est.*	*SE*	*z*	*p*	*CI lower*	*CI upper*
*Diet food/carb restriction regressed on:*						
MPS self-oriented perfectionism	**0.13**	**0.07**	**2.44**	**0.015**	**0.033**	**0.302**
ASI3 physical concerns	− 0.01	0.16	− 0.10	0.924	− 0.334	0.303
ASI3 cognitive concerns	0.15	0.16	0.93	0.354	− 0.16	0.447
ASI3 social concerns	− 0.20	0.15	− 1.42	0.157	− 0.492	0.079
BICI dysmorphic appearance concerns	0.20	0.16	1.37	0.170	− 0.093	0.526
BICI symptom interference	0.07	0.17	0.44	0.661	− 0.258	0.407
DERS18 awareness	− 0.03	0.09	− 0.43	0.666	− 0.218	0.139
DERS18 clarity	− **0.25**	**0.13**	− **2.31**	**0.021**	− **0.542**	− **0.044**
DERS18 nonacceptance	**0.22**	**0.10**	**2.25**	**0.025**	**0.03**	**0.436**
DERS18 strategies	− 0.33	0.20	− 1.80	0.072	− 0.767	0.033
DERS18 goals	0.08	0.10	0.85	0.394	− 0.115	0.293
DERS18 impulse	0.11	0.11	1.00	0.317	− 0.105	0.325
*R*^2^	0.13					
*Pressure to eat regressed on:*						
MPS self-oriented perfectionism	0.06	0.07	1.02	0.307	− 0.063	0.201
ASI3 physical concerns	− 0.22	0.17	− 1.33	0.184	− 0.562	0.108
ASI3 cognitive concerns	0.23	0.14	1.37	0.172	− 0.084	0.469
ASI3 social concerns	0.06	0.14	0.40	0.692	− 0.215	0.324
BICI dysmorphic appearance concerns	0.11	0.16	0.59	0.552	− 0.22	0.412
BICI symptom interference	− 0.08	0.20	− 0.39	0.697	− 0.462	0.309
DERS18 awareness	0.04	0.08	0.53	0.597	− 0.113	0.197
DERS18 clarity	0.13	0.11	1.09	0.276	− 0.099	0.346
DERS18 nonacceptance	0.17	0.09	1.67	0.095	− 0.027	0.341
DERS18 strategies	− 0.19	0.19	− 0.93	0.351	− 0.54	0.192
DERS18 goals	0.12	0.10	1.11	0.268	− 0.081	0.291
DERS18 impulse	− 0.08	0.12	− 0.59	0.555	− 0.292	0.157
*R*^2^	0.11					
*Desire for thinness regressed on:*						
MPS self-oriented perfectionism	0.06	0.04	1.56	0.119	− 0.018	0.155
ASI3 physical concerns	− 0.01	0.10	− 0.13	0.901	− 0.203	0.179
ASI3 cognitive concerns	− 0.02	0.10	− 0.21	0.833	− 0.206	0.166
ASI3 SOCIAL concerns	− 0.06	0.10	− 0.58	0.565	− 0.244	0.133
BICI dysmorphic appearance concerns	**0.73**	**0.09**	**7.92**	**< .001**	**0.516**	**0.855**
BICI symptom interference	0.03	0.10	0.25	0.802	− 0.171	0.221
DERS18 awareness	− 0.09	0.06	− 1.57	0.117	− 0.197	0.022
DERS18 clarity	− 0.03	0.08	− 0.41	0.680	− 0.184	0.12
DERS18 nonacceptance	0.00	0.07	− 0.04	0.967	− 0.136	0.131
DERS18 strategies	0.06	0.12	0.46	0.648	− 0.179	0.288
DERS18 goals	− 0.05	0.06	− 0.66	0.507	− 0.169	0.084
DERS18 impulse	0.07	0.08	0.76	0.450	− 0.097	0.218
*R*^2^	0.55					
*Purging (EAT26_25) regressed on:*						
MPS self-oriented perfectionism	0.05	0.16	0.46	0.647	− 0.242	0.39
ASI3 physical concerns	0.16	0.33	0.68	0.498	− 0.427	0.878
ASI3 cognitive concerns	**0.51**	**0.21**	**2.60**	**0.009**	**0.137**	**0.974**
ASI3 social concerns	− **0.78**	**0.33**	− **2.65**	**0.008**	− **1.529**	− **0.228**
BICI dysmorphic appearance concerns	0.37	0.40	1.09	0.276	− 0.346	1.212
BICI symptom interference	0.10	0.44	0.27	0.790	− 0.744	0.978
DERS18 awareness	**0.24**	**0.12**	**2.68**	**0.007**	**0.084**	**0.54**
DERS18 clarity	− **0.44**	**0.25**	− **2.26**	**0.024**	− **1.046**	− **0.074**
DERS18 nonacceptance	0.18	0.20	1.10	0.271	− 0.169	0.603
DERS18 strategies	0.61	0.48	1.55	0.121	− 0.198	1.7
DERS18 goals	− 0.19	0.37	− 0.58	0.562	− 0.947	0.515
DERS18 impulse	− 0.25	0.28	− 1.00	0.317	− 0.822	0.266
*R*^2^	0.5					
*Binging (EAT26_4) regressed on:*						
MPS self-oriented perfectionism	− 0.05	0.09	− 0.74	0.458	− 0.243	0.11
ASI3 physical concerns	0.24	0.17	1.88	0.060	− 0.013	0.662
ASI3 cognitive concerns	0.20	0.15	1.43	0.154	− 0.082	0.518
ASI3 social concerns	− **0.54**	**0.18**	− **3.39**	**0.001**	− **0.969**	− **0.259**
BICI dysmorphic appearance concerns	**0.42**	**0.16**	**3.05**	**0.002**	**0.176**	**0.813**
BICI symptom interference	0.16	0.19	1.00	0.319	− 0.182	0.558
DERS18 awareness	− 0.02	0.10	− 0.29	0.774	− 0.232	0.173
DERS18 clarity	− 0.10	0.13	− 0.96	0.337	− 0.368	0.126
DERS18 nonacceptance	0.02	0.11	0.23	0.818	− 0.183	0.231
DERS18 strategies	0.28	0.21	1.67	0.095	− 0.061	0.755
DERS18 goals	− 0.13	0.13	− 1.18	0.237	− 0.401	0.099
DERS18 impulse	0.01	0.13	0.10	0.920	− 0.249	0.276
*R*^2^	0.39					

Bolded entries indicate significant results at or below the .05 level

Examining diet/carb restriction, individuals one standard deviation (*SD*) higher in self-oriented perfectionism were expected to be 13% of an *SD* higher in dieting/carb-restrictive behaviors. Moving to emotion dysregulation, higher levels of emotional nonacceptance were associated with greater diet/carb-restriction, whereas individuals one *SD* higher in lack of emotional clarity were expected to be 25% of an *SD lower* in restrictive eating behaviors.

Results for the desire for thinness outcome indicated that individuals one *SD* higher on dysmorphic appearance concerns were expected to be 73% of an *SD* higher in their reported desire to be thin.

Regarding the EAT-26 purging outcome, AS cognitive and social concerns subscales differentially predicted eating behaviors. Individuals one *SD* higher on cognitive concerns were expected to be 51% of an *SD* higher in propensities to purge. Whereas individuals one *SD* higher on social concerns were expected to be 78% of an *SD lower* in their propensities to purge. Similarly, emotion dysregulation subscales showed differential associations with purging.

Individuals one *SD* higher on lack of emotional awareness were expected to be 24% of an *SD* higher in purging tendencies. However, individuals one *SD* higher on lack of emotional clarity were expected to be 44% of an *SD lower* in their propensities to purge.

Results for the EAT-26 binging outcome show that individuals one *SD* higher on dysmorphic appearance concerns were expected to be 42% of an *SD* higher in their propensities to binge. However, AS social concerns exhibited a protective influence over binging. Individuals one *SD* higher on social concerns were expected to be 54% of an *SD lower* in their tendencies to binge.

## Discussion

Results from this study support our initial hypotheses and provide some insight into the differential associations among psychosocial constructs and DE cognitions/behaviors. Anxiety sensitivity social concerns exhibited a protective pathway on DE, as individuals with higher concerns about what others think of their anxiety, showed lower tendencies to engage in binging and purging behaviors. Essentially, the influence of perceptions of what others think may provide a buffer that relates to lower purging/binging behavior. Perhaps the concern for what others think carries over from anxiety to eating behaviors [53]. These findings contrast with previous research in severe ED samples, where social concerns mediated by experiential avoidance predicted higher ED psychopathology [66]. Conversely, cognitive concerns of AS demonstrated a positive association with purging behaviors, consistent with previous research. Individuals high in fear of losing cognitive control may engage in maladaptive behaviors (i.e., purging) to regulate and avoid internal states [67].

Self-oriented perfectionism demonstrated a risk pathway for dieting/carb restriction, as found in previous literature with non-clinical samples [99, 100]. Holding extremely high personal standards may extend to one’s body and dietary choices. In contrast to previous research in both clinical and community samples, self-oriented perfectionism was not significantly associated with desire for thinness in our sample [34, 101, 102]. This non-significant association could be due to our sample characteristics, as average scores on the MPS and EAT-26 were below clinical thresholds. Additionally, in the Hispanic culture, ideals of beauty often prioritize curves and fuller figures over thinness, diverging from cultures that idealize thinness [103]. This cultural difference in beauty ideals could weaken the association between self-oriented perfectionism and desire for thinness in our predominantly Hispanic sample. It is also important to consider the complex interplay between variables, and certain factors may contrast or interact with each other, leading to varied results in terms of the significance of relationships.

Our results for dysmorphic appearance concerns were associated with higher binging tendencies, as found in previous research in community samples [26, 104]. Binge-eating might serve as a coping mechanism to temporarily alleviate the distress and negative emotions associated with body image dissatisfaction.

Lack of emotional awareness and emotional nonacceptance showed positive associations with purging tendencies and dieting/carb restriction respectively. Lack of emotional awareness has been linked with DE behaviors [105], including purging [106] in community samples. Individuals with problems paying attention and acknowledging their emotions may engage in maladaptive eating behaviors like purging, as a means to escape and avoid emotional discomfort. Purging may provide an immediate sense of emotional relief and control for individuals with difficulty recognizing and understanding their emotions. Similarly, emotional nonacceptance has been associated with ED psychopathology including dieting/carb restriction in college samples [107, 108]. Difficulty accepting and tolerating one’s emotions, which usually comes along with negative judgment of one’s emotions as unacceptable, may lead individuals to engage in dieting and carb-restrictive behaviors as a mechanism to regain a sense of control. By rigidly controlling their food intake, individuals may believe they are exerting control over their emotions as well.

Lack of emotional clarity showed a protective or buffering influence on dieting/carb restriction and purging tendencies. This inability to recognize and understand one’s emotions has been described as a complex dimension of emotion dysregulation [109]. Emotional clarity demonstrates inconsistent patterns in the literature but has been established as a risk factor for psychopathology [45, 110, 111]. Its negative association with EDs outcomes in this study is novel and requires further examination. Individuals who have less clarity over their emotional experience may also have fewer concerns or motivation to restrict their diet/carbs. Lack of clarity may drive individuals to avoid or suppress their emotions, which in turn reduces the emotional distress that often triggers the emotionally driven pathological eating behaviors (i.e., purging and dieting/carb restrictive behaviors). In other words, individuals who are not clear on what emotions they are experiencing may diminish their experience of emotion, and in turn, be less likely to rely on maladaptive behaviors to cope with distress. Interestingly, none of the emotion dysregulation subscales showed significant associations with binging, which contradicts extensive literature in both individuals with EDs and community samples [112, 113]. This could be due to measurement issues, as binging was measured through a single-item indicator that may not have fully captured the complexity and nuances of binge eating episodes, potentially leading to an underestimation of their association with emotion dysregulation subscales. Additionally, it is important to consider the developmental stage of the participants, as the relationship between emotion dysregulation and binging becomes more pronounced in older populations or in clinical samples [114] where individuals may have developed more maladaptive coping mechanisms. Another explanation could be the role of emotional eating, as higher levels of emotion dysregulation have been associated with greater emotional eating [115], which in turn, results in binge-eating. Further, previous longitudinal research in a clinical sample found no association between participant’s emotion dysregulation levels and their binging frequency [116]. Similarly, Peterson et al. [117], found that improvements in emotion regulation were not immediately linked to changes in binge eating frequency but showed an association at a 4-month follow-up. This suggests that while learning better ways to manage emotions might have an immediate impact on thoughts related to EDs, its effect on binge eating behavior might take longer to become apparent. Ultimately, these revealing differential pathways should be further explored, and prevention/intervention efforts should address different aspects of emotion dysregulation to prevent maladaptive DE coping strategies.

It is important to mention that none of our predictors was significantly associated with the pressure to eat outcome. These findings could be explained by the complex interplay of factors included in our model. Further, it is important to acknowledge that certain questions from the original EAT-26 did not load into our proposed 4-factor model, resulting in our refined pressure to eat outcome comprising only three items. This outcome measure may not align with the predictors in our model, which makes conceptual sense considering that neither body dysmorphia nor emotional regulation strategies appeared directly related to perceived pressure from others to eat. In addition, our sample characteristics could be associated with the non-significant associations. Our sample’s average score on the EAT-26 was below clinical cutoffs, and individuals may not experience significant pressure from others to eat, which results in little variability in the outcome variable, making it difficult to detect significant associations with your predictors.

We emphasize that the factors we included in our analyses have shown to be related to one another, and to ED outcomes. As a result, we anticipated that there would be some correlation, shared variance associated with the factors in the model. Therefore, to appropriately handle these shared variances, we adopted a SEM approach, that allowed us to comprehensively address the intercorrelations among variables by simultaneously modeling these relationships. Findings from our model provide a nuanced understanding of the complex dynamics among ED risk and resilience factors.

The present study has several limitations. Our cross-sectional design restricts us from establishing causal relationships between psychosocial factors and DE outcomes. The single-item indicators for binging and purging constructs may have introduced measurement error and lowered the precision of our estimates in these parts of the model. Additionally, self-report measures may have introduced bias that may have influenced the observed indicators and potentially introduced measurement error in latent constructs. Further, despite relying on a novel factorial structure for the EAT-26 that performs more adequately in our sample, the diet foods/carbs restriction and pressure to eat subscales achieved low/acceptable internal reliability. This suggests that our two subscales might not fully capture the underlying constructs being measured, potentially affecting the validity of our findings related to those subscales.

It is also important to note that self-reported scores across all constructs were below clinical thresholds, including the EAT-26. This limits the generalizability of our findings to clinically diagnosed ED populations, and points to a line for future research. Our study relied on convenience sampling from university students, which resulted in a predominantly female sample (70%), with most identifying as Hispanic (60%). However, our sample was representative of the larger population from which it was drawn, and its uniqueness allowed us to approach DE in a more inclusive and diverse way, and advance understanding of these pathologies as they differentially affect and present in those from understudied and underrepresented groups. The differential results observed in the study could indeed be attributed, at least in part, to the unique cultural composition of our sample, highlighting the importance of considering cultural factors in understanding and addressing DE. Traditionally, most research and treatment on EDs has focused on middle-class White women, leaving other vulnerable groups including those from minority backgrounds largely overlooked [13, 14, 118, 119]. Thus, it is imperative that future studies address the call for diversity in EDs research proposed by Halbeisen et al. [120], including non-SWAG (Skinny White Affluent Girls) stereotyped samples. Eating pathologies are rising across diverse understudied populations [120, 121]. Diversity and culturally oriented research is the only path to understand the causes and manifestations of EDs across underrepresented populations, and help develop more appropriate prevention/intervention efforts aligned with prevalence rates in the current society.

## Conclusions

The present study adopts a multivariate approach to examine latent variables in a complex SEM, with the aim to approximate more closely to the nuances of EDs risk. Our model demonstrates the complexity of eating pathologies and exposes how psychosocial factors are differently associated with DE outcomes, showing both risk and resilience pathways. Effective transdiagnostic prevention/intervention is dependent upon improved clarity in factors that constitute risk and resilience, and awareness of developmental and cultural influences in EDs/DE emergence.

## Data Availability

The data that support the findings of this study are available from the corresponding author upon reasonable request.

## Footnotes

1 Symptoms vary by degree ranging from no symptoms to diagnosed clinical EDs [9], see also [20], [122]. This conceptualization of EDs represents their dimensional nature and is therefore most appropriate for capturing variability in predictors and outcomes [123].

2 Hewitt et al. [78] proposed a model encompassing: (1) self-oriented perfectionism (e.g., perfectionistic behaviors directed to oneself), (2) other-oriented perfectionism (e.g., beliefs and expectations about the capacities of others), and (3) socially perceived perfectionism (e.g., the perceived need to achieve standards and expectations set by others) [78].

3 Physical concerns refer to the fear of physical symptoms associated with anxiety; cognitive concerns refer to the fear of losing control of one’s cognitions; and social concerns refer to the fear of one’s anxiety symptoms being socially observable [57].

4 The factor structure of the EAT-26 was thoroughly examined in the current sample. The original 3-factor structure of the EAT-26 demonstrated unacceptable model fit across most indices (*CFI* = 0.85; *TLI* = 0.83; *RMSEA* = 0.07; *SRMR* = 0.14). An exploratory factor analysis (EFA) was conducted in MPlus, using WLSMV estimation. We extracted 1- through 7-factor models and followed statistical and substantive criteria to arrive to the 4-factor solution used in the present research. The 4-factor EFA achieved high model fit (*CFI* = 0.98; *TLI* = 0.98; *RMSEA* = 0.03; *SRMR* = 0.05). Lastly, to assess the model fit of this new 4-factor solution in our data we conducted a CFA, which achieved high model fit (*CFI* = 0.97; *TLI* = 0.96; *RMSEA* = 0.06; *SRMR* = 0.09). Both CFA analyses were conducted in lavaan using WLSMV estimation. Readers interested in a more detailed explanation of the analyses are referred to reference [53].

5 For model specification and the appropriate treatment of categorical variables, we estimated our model in different steps: first, we estimated the model through maximum likelihood with robust standard errors, and handled missing data with *FIML* [93]; second, we obtained the covariance matrix of the observed variables from this model, extracting the ordered variable names; and finally, we re-estimated our SEM model incorporating the ordered argument to make sure that the model properly accounted for the categorical nature of our observed variables.

## References

[1] Iwajomo T, Bondy SJ, De Oliveira C, Colton P, Trottier K, Kurdyak P (2021). Excess mortality associated with eating disorders: population-based cohort study. Br J Psychiatry.

[2] Arcelus J, Mitchell AJ, Wales J, Nielsen S (2011). Mortality rates in patients with anorexia nervosa and other eating disorders: a meta-analysis of 36 studies. Arch Gen Psychiatry.

[3] Galmiche M, Déchelotte P, Lambert G, Tavolacci MP (2019). Prevalence of eating disorders over the 2000–2018 period: a systematic literature review. Am J Clin Nutr.

[4] Deloitte Access Economics. The social and economic cost of eating disorders in the United States of America: a report for the strategic training initiative for the prevention of eating disorders and the Academy for Eating Disorders. 2020. https://www.hsph.harvard.edu/striped/report-economic-costs-of-eating-disorders/. Accessed 03 Oct 2023.

[5] Bakalar JL, Shank LM, Vannucci A, Radin RM, Tanofsky-Kraff M (2015). Recent advances in developmental and risk factor research on eating disorders. Curr Psychiatry Rep.

[6] Booij L, Steiger H (2020). Applying epigenetic science to the understanding of eating disorders: a promising paradigm for research and practice. Curr Opin Psychiatry.

[7] Barakat S (2023). Risk factors for eating disorders: findings from a rapid review. J Eat Disord.

[8] Fox JRE, Power MJ (2009). Eating disorders and multi-level models of emotion: an integrated model. Clin Psychol Psychother Int J Theory Pract.

[9] Tylka TL, Subich LM (2004). Examining a multidimensional model of eating disorder symptomatology among college women. J Couns Psychol.

[10] Leung F, Geller J, Katzman M (1996). Issues and concerns associated with different risk models for eating disorders. Int J Eat Disord.

[11] Schmidt U (2003). Aetiology of eating disorders in the 21st century. Eur Child Adolesc Psychiatry.

[12] Stice E (2002). Risk and maintenance factors for eating pathology: a meta-analytic review. Psychol Bull.

[13] Rodgers RF, Berry R, Franko DL (2018). Eating disorders in ethnic minorities: an update. Curr Psychiatry Rep.

[14] Cheng ZH, Perko VL, Fuller-Marashi L, Gau JM, Stice E (2019). Ethnic differences in eating disorder prevalence, risk factors, and predictive effects of risk factors among young women. Eat Behav.

[15] Udo T, Grilo CM (2018). Prevalence and correlates of DSM-5–defined eating disorders in a nationally representative sample of U.S. adults. Biol Psychiatry.

[16] Marques L (2011). Comparative prevalence, correlates of impairment, and service utilization for eating disorders across US ethnic groups: implications for reducing ethnic disparities in health care access for eating disorders. Int J Eat Disord.

[17] William F. 2000 US census and census population estimates, 2020.

[18] D. S. American Psychiatric Association and A. P. Association (2013). Diagnostic and statistical manual of mental disorders: DSM-5.

[19] Sanford-Martens TC, Davidson MM, Yakushko OF, Martens MP, Hinton P (2005). Clinical and subclinical eating disorders: an examination of collegiate athletes. J Appl Sport Psychol.

[20] Shisslak CM, Crago M, Estes LS (1995). The spectrum of eating disturbances. Int J Eat Disord.

[21] Stice E, Marti CN, Rohde P (2013). Prevalence, incidence, impairment, and course of the proposed DSM-5 eating disorder diagnoses in an 8-year prospective community study of young women. J Abnorm Psychol.

[22] Combs JL, Pearson CM, Zapolski TCB, Smith GT (2013). Preadolescent disordered eating predicts subsequent eating dysfunction. J Pediatr Psychol.

[23] Fairburn CG, Cooper Z, Doll HA, Davies BA (2005). Identifying dieters who will develop an eating disorder: a prospective, population-based study. Am J Psychiatry.

[24] Martinez MA, Craighead LW (2015). Toward person(ality)-centered treatment: How consideration of personality and individual differences in anorexia nervosa may improve treatment outcome. Clin Psychol Sci Pract.

[25] Bardone-Cone AM (2007). Perfectionism and eating disorders: current status and future directions. Clin Psychol Rev.

[26] Boone L, Soenens B, Luyten P (2014). When or why does perfectionism translate into eating disorder pathology? A longitudinal examination of the moderating and mediating role of body dissatisfaction. J Abnorm Psychol.

[27] Brown AJ, Parman KM, Rudat DA, Craighead LW (2012). Disordered eating, perfectionism, and food rules. Eat Behav.

[28] Santonastaso P, Friederici S, Favaro A (1999). Full and partial syndromes in eating disorders: a 1-year prospective study of risk factors among female students. Psychopathology.

[29] Shafran R, Mansell W (2001). Perfectionism and psychopathology: a review of research and treatment. Clin Psychol Rev.

[30] Vohs KD, Heatherton TF, Herrin M (2001). Disordered eating and the transition to college: a prospective study. Int J Eat Disord.

[31] Cockell SJ (2002). Trait and self-presentational dimensions of perfectionism among women with anorexia nervosa. Cognit Ther Res.

[32] Sassaroli S, Romero Lauro LJ, Maria Ruggiero G, Mauri MC, Vinai P, Frost R (2008). Perfectionism in depression, obsessive-compulsive disorder and eating disorders. Behav Res Ther.

[33] Dickie L, Surgenor LJ, Wilson M, McDowall J (2012). The structure and reliability of the Clinical Perfectionism Questionnaire. Pers Individ Dif.

[34] Rivière J, Douilliez C (2017). Perfectionism, rumination, and gender are related to symptoms of eating disorders: a moderated mediation model. Personal Individ Differ.

[35] Barnett MD, Sharp KJ (2016). Maladaptive perfectionism, body image satisfaction, and disordered eating behaviors among U.S. college women: the mediating role of self-compassion. Personal Individ Differ.

[36] Davis C (1997). Normal and neurotic perfectionism in eating disorders: an interactive model. Int J Eat Disord.

[37] Brosof LC, Levinson CA (2017). Social appearance anxiety and dietary restraint as mediators between perfectionism and binge eating: a six month three wave longitudinal study. Appetite.

[38] Czepiel D, Koopman HM (2021). Does physical appearance perfectionism predict disordered dieting?. Curr Psychol.

[39] Egan SJ, Wade TD, Shafran R (2011). Perfectionism as a transdiagnostic process: a clinical review. Clin Psychol Rev.

[40] Lloyd S, Fleming C, Tchanturia K, Tchanturia K (2015). Perfectionism short format group for inpatients. Brief group psychotherapy for eating disorders: inpatient protocols.

[41] Thompson RA (1994). Emotion regulation: a theme in search of definition. Monogr Soc Res Child Dev.

[42] Gross JJ, Lewis M, Haviland-Jones JM, Barrett LF (2008). Emotion regulation. Handbook of emotions.

[43] D’Agostino A, Covanti S, Rossi Monti M, Starcevic V (2017). Reconsidering emotion dysregulation. Psychiatr Q.

[44] Beauchaine TP, Gatzke-Kopp L, Mead HK (2007). Polyvagal theory and developmental psychopathology: emotion dysregulation and conduct problems from preschool to adolescence. Biol Psychol.

[45] Monell E, Clinton D, Birgegård A (2018). Emotion dysregulation and eating disorders—associations with diagnostic presentation and key symptoms. Int J Eat Disord.

[46] Brockmeyer T (2014). Difficulties in emotion regulation across the spectrum of eating disorders. Compr Psychiatry.

[47] Svaldi J, Griepenstroh J, Tuschen-Caffier B, Ehring T (2012). Emotion regulation deficits in eating disorders: a marker of eating pathology or general psychopathology?. Psychiatry Res.

[48] Corstorphine E (2006). Cognitive–emotional–behavioural therapy for the eating disorders: working with beliefs about emotions. Eur Eat Disord Rev.

[49] Fox JRE (2009). A qualitative exploration of the perception of emotions in anorexia nervosa: a basic emotion and developmental perspective. Clin Psychol Psychother.

[50] Lavender JM, Wonderlich SA, Engel SG, Gordon KH, Kaye WH, Mitchell JE (2015). Dimensions of emotion dysregulation in anorexia nervosa and bulimia nervosa: a conceptual review of the empirical literature. Clin Psychol Rev.

[51] Haynos AF, Fruzzetti AE (2011). Anorexia nervosa as a disorder of emotion dysregulation: evidence and treatment implications. Clin Psychol Sci Pract.

[52] Barlow DH, Allen LB, Choate ML (2004). Toward a unified treatment for emotional disorders. Behav Ther.

[53] Bazo Perez M, Hayes T, Frazier L (2023). Beyond generalized anxiety: the association of anxiety sensitivity with disordered eating. J Eat Disord.

[54] Frazier LD, Waid LD (1999). Influences on anxiety in later life: the role of health status, health perceptions, and health locus of control. Aging Ment Health.

[55] Reiss S (1991). Expectancy model of fear, anxiety, and panic. Clin Psychol Rev.

[56] Reiss S, Peterson RA, Gursky DM, McNally RJ (1986). Anxiety sensitivity, anxiety frequency and the prediction of fearfulness. Behav Res Ther.

[57] Taylor S (2007). Robust dimensions of anxiety sensitivity: development and initial validation of the anxiety sensitivity index-3. Psychol Assess.

[58] Barlow DH. The nature of anxiety: anxiety, depression, and emotional disorders. In: Chronic anxiety: generalized anxiety disorder and mixed anxiety-depression. New York: Guilford Press; 1991. p. 1–28.

[59] Schmidt NB, Zvolensky MJ, Maner JK (2006). Anxiety sensitivity: prospective prediction of panic attacks and axis I pathology. J Psychiatr Res.

[60] Mennin DS, Heimberg RG, Turk CL, Fresco DM (2005). Preliminary evidence for an emotion dysregulation model of generalized anxiety disorder. Behav Res Ther.

[61] Craske MG (2012). Transdiagnostic treatment for anxiety and depression. Depress Anxiety.

[62] Wildes JE, Marcus MD (2011). Development of emotion acceptance behavior therapy for anorexia nervosa: a case series. Int J Eat Disord.

[63] Anestis MD, Holm-Denoma JM, Gordon KH, Schmidt NB, Joiner TE (2008). The role of anxiety sensitivity in eating pathology. Cognit Ther Res.

[64] Lillis J, Hayes SC, Levin ME (2011). Binge eating and weight control: the role of experiential avoidance. Behav Modif.

[65] Wildes JE, Ringham RM, Marcus MD (2010). Emotion avoidance in patients with anorexia nervosa: initial test of a functional model. Int J Eat Disord.

[66] Espel-Huynh HM, Muratore AF, Virzi N, Brooks G, Zandberg LJ (2019). Mediating role of experiential avoidance in the relationship between anxiety sensitivity and eating disorder psychopathology: a clinical replication. Eat Behav.

[67] Fulton JJ, Lavender JM, Tull MT, Klein AS, Muehlenkamp JJ, Gratz KL (2012). The relationship between anxiety sensitivity and disordered eating: the mediating role of experiential avoidance. Eat Behav.

[68] Stice E, Shaw HE (2002). Role of body dissatisfaction in the onset and maintenance of eating pathology. J Psychosom Res.

[69] Higgins ET (1989). Self-discrepancy theory: What patterns of self-beliefs cause people to suffer?.

[70] Higgins ET (1987). Self-discrepancy: a theory relating self and affect. Psychol Rev.

[71] Biolcati R, Mancini G, Villano P (2020). ‘And yet I’m an adult now’. The influence of parental criticism on women’s body satisfaction/dissatisfaction during emerging adulthood. Int J Adolesc Youth.

[72] Fairburn CG (2008). Cognitive behavior therapy and eating disorders.

[73] Thompson JK, Heinberg LJ, Altabe M, Tantleff-Dunn S (1999). Exacting beauty: theory, assessment, and treatment of body image disturbance.

[74] Yu K, Perez M (2019). The association between maternal criticism and body dissatisfaction on disordered eating pathology across racial and ethnic groups. Cult Divers Ethnic Minor Psychol.

[75] Brausch AM, Muehlenkamp JJ (2007). Body image and suicidal ideation in adolescents. Body Image.

[76] Orbach I, Mikulincer M (1998). The body investment scale: construction and validation of a body experience scale. Psychol Assess.

[77] Palmeroni N, Luyckx K, Verschueren M, Claes L (2020). Body dissatisfaction as a mediator between identity formation and eating disorder symptomatology in adolescents and emerging adults. Psychol Belg.

[78] Hewitt PL, Flett GL, Turnbull-Donovan W, Mikail SF (1991). The multidimensional perfectionism scale: reliability, validity, and psychometric properties in psychiatric samples. Psychol Assess J Consult Clin Psychol.

[79] Victor SE, Klonsky ED (2016). Validation of a brief version of the difficulties in emotion regulation scale (DERS-18) in five samples. J Psychopathol Behav Assess.

[80] Littleton HL, Axsom D, Pury CLS (2005). Development of the body image concern inventory. Behav Res Ther.

[81] Garner D, Olmsted M, Bohr Y, Garfinkel P (1982). The eating attitudes test: psychometric features and clinical correlates. Psychol Med.

[82] Park J, Beaudet MP (2007). Eating attitudes and their correlates among Canadian women concerned about their weight. Eur Eat Disord Rev.

[83] Papini NM (2022). Psychometric properties of the 26-item eating attitudes test (EAT-26): an application of Rasch analysis. J Eat Disord.

[84] Desai MN, Miller WC, Staples B, Bravender T (2008). Risk factors associated with overweight and obesity in college students. J Am Coll Health.

[85] Rivas T, Bersabé R, Jiménez M, Berrocal C (2010). The eating attitudes test (EAT-26): RELIABILITY and validity in Spanish female samples. Span J Psychol.

[86] Rogoza R, Brytek-Matera A, Garner DM (2016). Analysis of the EAT-26 in a non-clinical sample. Arch Psychiatry Psychother.

[87] Belon KE, Smith JE, Bryan AD, Lash DN, Winn JL, Gianini LM (2011). Measurement invariance of the Eating Attitudes Test-26 in Caucasian and Hispanic women. Eat Behav.

[88] Khaled SM, Kimmel L, Le Trung K (2018). Assessing the factor structure and measurement invariance of the eating attitude test (EAT-26) across language and BMI in young Arab women. J Eat Disord.

[89] Spivak-Lavi Z, Peleg O, Tzischinsky O, Stein D, Latzer Y (2021). Differences in the factor structure of the eating attitude test-26 (Eat-26) in different cultures in Israel: Jews, Muslims, and Christians. Nutrients.

[90] Schaefer LM (2019). Gender-based differential item functioning in measures of eating pathology. Int J Eat Disord.

[91] Rosseel Y (2012). **lavaan**: an
* R
* package for structural equation modeling. J Stat Softw.

[92] Bunn A, et al. dplR: Dendrochronology program library in R, v 1.6. 4; 2016.

[93] Arbuckle JL, Marcoulides GA, Schumacker RE (1996). Full information estimation in the presence of incomplete data. Advanced structural equation modeling: issues and techniques.

[94] Bentler PM (1990). Comparative fit indexes in structural models. Psychol Bull.

[95] Tucker LR, Lewis C (1973). A reliability coefficient for maximum likelihood factor analysis. Psychometrika.

[96] Steiger JH, Lind JC. Statistically based tests for the number of common factors. In: Psychometric society annual meeting, Iowa City; 1980.

[97] Steiger JH (2016). Notes on the Steiger–Lind (1980) Handout. Struct Equ Model.

[98] Jöreskog KG, Sörbom D (1993). LISREL 8.

[99] Joyce F, Watson HJ, Egan SJ, Kane RT (2012). Mediators between perfectionism and eating disorder psychopathology in a community sample. Eat Behav.

[100] Fitzsimmons-Craft EE, Bardone-Cone AM, Brownstone LM, Harney MB (2012). Evaluating the roles of anxiety and dimensions of perfectionism in dieting and binge eating using weekly diary methodology. Eat Behav.

[101] Dickie L, Wilson M, McDowall J, Surgenor LJ (2012). What components of perfectionism predict drive for thinness?. Eat Disord.

[102] Lilenfeld LRR, Wonderlich S, Riso LP, Crosby R, Mitchell J (2006). Eating disorders and personality: a methodological and empirical review. Clin Psychol Rev.

[103] Chamorro R, Flores-Ortiz Y (2000). Acculturation and disordered eating patterns among Mexican American women. Int J Eat Disord.

[104] Pennesi J-L, Wade TD (2016). A systematic review of the existing models of disordered eating: Do they inform the development of effective interventions?. Clin Psychol Rev.

[105] Bullock AJ, Goldbacher EM (2021). Interoceptive awareness and emotional eating in college women: the role of appetite and emotional awareness. J Am Coll Health.

[106] Laghi F, Pompili S, Bianchi D, Lonigro A, Baiocco R (2021). Drunkorexia: an examination of the role of theory of mind and emotional awareness among adolescents. Dev Neuropsychol.

[107] Leppanen J, Brown D, McLinden H, Williams S, Tchanturia K (2022). The role of emotion regulation in eating disorders: a network meta-analysis approach. Front Psychiatry.

[108] Mikhail ME, Kring AM (2019). Emotion regulation strategy use and eating disorder symptoms in daily life. Eat Behav.

[109] Lischetzke T, Eid M, Robinson M, Eid M (2017). The functionality of emotional clarity: a process-oriented approach to understanding the relation between emotional clarity and well-being. The happy mind: cognitive contributions to well-being.

[110] Vuillier L, May L, Greville-Harris M, Surman R, Moseley RL (2021). The impact of the COVID-19 pandemic on individuals with eating disorders: the role of emotion regulation and exploration of online treatment experiences. J Eat Disord.

[111] Benzerouk F, Djerada Z, Bertin E, Barrière S, Gierski F, Kaladjian A (2020). Contributions of emotional overload, emotion dysregulation, and impulsivity to eating patterns in obese patients with binge eating disorder and seeking bariatric surgery. Nutrients.

[112] Braden A (2023). Eating when depressed, anxious, bored, or happy: an examination in treatment-seeking adults with overweight/obesity. Appetite.

[113] Leehr EJ, Krohmer K, Schag K, Dresler T, Zipfel S, Giel KE (2015). Emotion regulation model in binge eating disorder and obesity—a systematic review. Neurosci Biobehav Rev.

[114] Mangweth-Matzek B (2014). Prevalence of eating disorders in middle-aged women. Int J Eat Disord.

[115] Michopoulos V, Powers A, Moore C, Villarreal S, Ressler KJ, Bradley B (2015). The mediating role of emotion dysregulation and depression on the relationship between childhood trauma exposure and emotional eating. Appetite.

[116] Bodell LP (2019). Longitudinal associations between emotion regulation skills, negative affect, and eating disorder symptoms in a clinical sample of individuals with binge eating. Eat Behav.

[117] Peterson CB (2017). The effects of psychotherapy treatment on outcome in bulimia nervosa: examining indirect effects through emotion regulation, self-directed behavior, and self-discrepancy within the mediation model. Int J Eat Disord.

[118] Sonneville KR, Lipson SK (2018). Disparities in eating disorder diagnosis and treatment according to weight status, race/ethnicity, socioeconomic background, and sex among college students. Int J Eat Disord.

[119] Veach C, Munsell SE (2022). Eating disorders and the experiences of culturally diverse groups. Educ Res Theory Pract.

[120] Halbeisen G, Brandt G, Paslakis G (2022). A plea for diversity in eating disorders research. Front Psychiatry.

[121] Qian J (2022). An update on the prevalence of eating disorders in the general population: a systematic review and meta-analysis. Eat Weight Disord.

[122] Johnson MD (1994). Disordered eating in active and athletic women. Clin Sports Med.

[123] Luo X, Donnellan MB, Burt SA, Klump KL (2016). The dimensional nature of eating pathology: evidence from a direct comparison of categorical, dimensional, and hybrid models. J Abnorm Psychol.

